# Impact of perioperative COVID-19 infection on postoperative complication in cesarean section using Korean National Health insurance data

**DOI:** 10.1038/s41598-024-66901-5

**Published:** 2024-07-11

**Authors:** Hyo Jin Kim, EunJin Ahn, Gunn Hee Kim, Ji-Hyun Noh, Si Ra Bang

**Affiliations:** 1 Department of Anesthesiology and Pain Medicine, Chung-Ang University Gwangmyeong Hospital, Chung-Ang University College of Medicine, 110, Deokan-Ro, Gwangmyeong-Si, Gyeonggi-Do Republic of Korea; 2 Department of Anesthesiology and Pain Medicine, National Medical Center, Seoul, 04564 Republic of Korea; 3 Department of Obstetrics and Gynecology, Inje University Sanggye Paik Hospital, Seoul, 01757 Republic of Korea

**Keywords:** COVID-19, Cesarean section, Anesthesia, Epidemiology, Diseases, Medical research

## Abstract

The vulnerability during pregnancy has raised concerns about the potential impact of COVID-19 on obstetric anesthesia, an essential aspect of maternal care during cesarean section procedures. To evaluate the influence of COVID-19 infection on obstetric anesthesia during cesarean section, we analyzed the data from Korean National Health Insurance System (NHIS). This retrospective study utilized data from Korean NHIS. We included patients admitted with operation codes specific to cesarean section between January 1, 2020, and December 31, 2021. We classified patients into a COVID (+) group with a diagnosis code (U071) 30 days around surgery and a COVID (−) group without the code in the same period. The primary outcome was 30-day mortality that was defined as death within 30 days of admission due to any causes. Secondary outcomes were pulmonary complications (pneumonia, acute respiratory distress syndrome [ARDS], pulmonary thromboembolism [PTE], or unexpected postoperative mechanical ventilation), ICU admission, cardiac arrest, myocardial infarction [MI], other thromboembolic events, surgical site infection, sepsis, acute renal failure [ARF], and hepatic failure. Among 75,268 patients who underwent cesarean section, 107 had a COVID-19 diagnosis code, while 75,161 did not. After 1:4 propensity score matching (PSM), 535 patients were included in each group. 30-day mortality showed no significant differences between the two groups both before and after PSM. The COVID (+) group demonstrated significantly elevated rates of pneumonia, ARDS, PTE, and surgical site infection both before and after PSM. Hospital length of stay and admission costs were also significantly longer and higher, respectively, in the COVID (+) group before and after PSM. In subgroup analysis, no differences were observed in mortality and postoperative complications based on the anesthesia method after matching. COVID-19 infection is associated with increased rates of postoperative complications, including pneumonia, ARDS, PTE, surgical site infection, longer hospital stays, and increased admission costs, in patients who underwent cesarean section.

## Introduction

The coronavirus disease 2019 (COVID-19) pandemic, caused by the novel coronavirus severe acute respiratory syndrome coronavirus-2 (SARS-CoV-2), has had profound effects on healthcare systems worldwide, prompting significant attention to its impact on vulnerable populations, including pregnant individuals. Recent reviews have highlighted an increased risk of severe COVID-19 infection among pregnant patients, with higher rates of intensive care unit (ICU) admissions and mechanical ventilation compared to the general population^1,2^. This heightened vulnerability during pregnancy has raised concerns about the potential impact of COVID-19 on obstetric anesthesia, an essential aspect of maternal care during cesarean section procedures.

Understanding the impact of COVID-19 infection on obstetric anesthesia is of utmost importance for several reasons. Firstly, pregnant individuals have been identified as a potentially vulnerable group, with physiological changes during pregnancy potentially affecting disease severity and clinical outcomes^3–7^. Secondly, anesthesia plays a crucial role in ensuring safe cesarean section deliveries, and any alterations in anesthesia requirements due to COVID-19 infection may have implications for maternal and fetal well-being^8,9^. Moreover, investigating the association between COVID-19 infection and maternal mortality and pulmonary complications is vital for identifying potential risk factors and designing targeted interventions to mitigate adverse outcomes.

This study aims to evaluate the impact of COVID-19 infection on obstetric anesthesia in patients undergoing cesarean section procedures. To achieve this, we utilize a comprehensive dataset provided by the Korean National Health Insurance System (KNHI) and the National Health Insurance Sharing Service (NHIS), encompassing a large proportion of cesarean section operations performed in Korea between January 1, 2020, and December 31, 2021. The KNHI covers approximately 97% of the Korean population, ensuring the inclusion of a diverse and representative patient cohort. The study protocol received ethical approval from the Institutional Review Board of Inje University Seoul Paik Hospital, ensuring adherence to rigorous scientific and ethical standards.

Considering the global significance of the COVID-19 pandemic and the unique challenges it poses for maternal health, our study aims to contribute valuable insights into the interplay between COVID-19 infection and obstetric anesthesia. By shedding light on this crucial aspect of maternal care, our findings may inform healthcare professionals, policymakers, and researchers in their efforts to optimize the management of COVID-19 in pregnant individuals and improve overall maternal health outcomes.

## Methods

This study was approved by the Institutional Review Board of Inje University Seoul Paik Hospital (PAIK 2023-05-001). The requirement for informed consent was waived by the Institutional Review Board of Inje University Seoul Paik Hospital since we used de-identified administrative claims data. All methods were performed in accordance with the relevant guidelines and regulations.

### Study design

This retrospective study utilized data from the Korean NHIS, a singular mandatory national healthcare institution encompassing nearly the entire Korean population. Citizens are obliged to enroll in the NHIS, and claims submitted for reimbursement undergo review by the Health Insurance Review and Assessment Service. The dataset for our study was extracted from the National Health Information Database (NHID), established by the NHIS. The NHID is a publicly accessible database containing comprehensive information on healthcare utilization, health screenings, sociodemographic variables, and mortality for the entire population of South Korea, spanning the years 2020 to 2021. Access to the NHID is granted to researchers with approved study protocols by the official review committee.

Healthcare claims data, in this context, pertains to information derived from medical care benefit statements submitted by healthcare institutions for reimbursement from the NHIS. This dataset encompasses details on medical institutions, patients' personal information, International Classification of Diseases, 10th revision (ICD-10) codes, medical history (tests, procedures, and surgeries), prescriptions, and associated costs. The National Health Insurance Sharing Service (NHISS), operated by the NHIS, facilitates policy and academic research by providing support through the dissemination of public health information^10^.

For all (customized) data, the provided variables are restricted to the research purpose, with only results corresponding to the research design made available, and raw data remains inaccessible. The concept of data ownership is not applicable, and customized data analysis can be conducted either by visiting analysis centers operated by respective institutions or, in some cases, remotely accessing the data for analysis within private laboratories. National data meeting researcher-specified conditions are accessible to researchers^11^.

### Participant

We included all patients who underwent cesarean section in hospitals in Korea between January 1, 2020, and December 31, 2021. The major inclusion criterion was admission with a specific operation code for cesarean section surgery (R4514, R4516, R4517, R4518, R4519, R4520, R4507, R4508, R4509, R4510, R5001, R5002). Patients with a diagnosis of pneumonia within 1 year were excluded. Also, we excluded patients with missing data.

### Variables and outcome

Patients were categorized into two groups according to COVID-19 diagnosis code (U071). The COVID (+) group was defined as patients with a COVID-19 diagnosis code (U071) within 30 days before or after surgery. The COVID (−) group consists of patients with no COVID-19 diagnosis code (U071) within 30 days before or after surgery. Demographic characteristics, such as sex, age, region, and economic level were recorded. Additionally, data on COVID-19 vaccination status, diagnosis, surgical procedures, anesthesia methods (general or regional), and American Society of Anesthesiologists (ASA) physical status classification were documented. Information on the length of hospital stay and the type of hospital (clinic, general hospital, tertiary care hospital) was also captured. Other recorded details encompassed admission to the ICU, the duration of ICU stay, and the application of mechanical ventilation. The dataset included emergency surgery status, and complications such as venous thromboembolism, pulmonary thromboembolism (PTE), pneumonia, and acute respiratory distress syndrome (ARDS). Furthermore, the patient's survival status, date of death, cause of death, and comorbid conditions has been included. Comorbidities were assessed using the Charlson Comorbidity Index (CCI)^12^ and Elixhauser Comorbidity Score (ECS)^13^, which classify patients' additional health conditions based on ICD diagnosis codes. These methods assign weights to specific comorbidities, reflecting their impact on in-hospital mortality. The total score offers a numerical measure of a patient's overall comorbidity burden, assisting in risk assessment and outcome prediction. This includes diagnoses such as congestive heart failure, cardiac arrhythmias, valvular disease, pulmonary circulation disorders, peripheral vascular disorders, uncomplicated hypertension, complicated hypertension, paralysis, other neurologic disorders, chronic pulmonary disease, uncomplicated diabetes mellitus, complicated diabetes mellitus, hypothyroidism, renal failure, liver disease, peptic ulcer disease, acquired immune deficiency syndrome, or human immunodeficiency virus infection, lymphoma, metastatic cancer, solid tumor without metastasis, rheumatoid arthritis, coagulopathy, obesity, weight loss, fluid and electrolyte disorders, blood loss anemia, deficiency anemia, alcohol abuse, drug abuse, psychoses, and depression. The hospital type was classified as clinics, hospitals, general hospitals, or tertiary hospitals, and was determined by the number of beds. Clinics had up to 29 inpatient beds, hospitals had a minimum of 30, general hospitals had a minimum of 100 with physician specialists, and tertiary hospitals indicated general hospitals that were approved to provide most types of advanced medical care with a minimum of 20 departments^14^.

The primary outcome was 30-day mortality, defined as death within 30 days of admission due to any cause. Secondary outcomes included pulmonary complications (pneumonia, ARDS, PTE, or unexpected postoperative mechanical ventilation), ICU admission, cardiac arrest, myocardial infarction (MI), other thromboembolic events, surgical site infection, sepsis, acute renal failure (ARF), and hepatic failure.

### Statistical analysis

We matched perioperative COVID (+) group and COVID (−) group in a 1:4 ratio by propensity score matching (PSM) via the caliper matching method to minimize selection bias and the difference in demographic characteristics and comorbidities between the two groups. The propensity scores, estimated through logistic regression analysis utilizing variables such as age (analyzed as continuous variable), CCI, ASA physical status classification 3 or higher, and comorbidities (hypertension, diabetes, liver and kidney disease) were employed for a greedy 1:4 matching. The standardized mean difference (SMD) was defined as less than 0.1 in absolute value (Supplementary Fig. 1). To enhance the quality of the matching, we used the Logit Propensity Score (LPS) as the matching criterion. The caliper, defined as the allowable tolerance for matching, was set to 0.1 times the standard deviation of the LPS. This caliper setting ensured that matched pairs were sufficiently similar in terms of their propensity scores, thereby improving the comparability of the groups.

In addition, logistic regression models, Chi-square tests, and T-tests were utilized. For the entire surgical cohort, PSM variables included age, gender, and CCI, ensuring that the SMD for all variables was below 0.1. Post-matching results showed 107 COVID (+) patients and 428 COVID (−) patients, totaling 535 surgical patients as the final study cohort.

The normal distribution of variables was evaluated via the Kolmogorov–Smirnov test or Shapiro–Wilk test. For pre-matching data, continuous variables were analyzed using the Wilcoxon rank-sum test, and categorical variables were compared using the Chi-square test or Fisher’s exact test. For post-matching, continuous variables were tested using paired t-test or Wilcoxon’s signed rank-sum test, and categorical variables were compared with the McNemar test or exact McNemar test. All statistical analyses used were two-sided, and the significance level was set at a P-value less than 0.05. R version 3.4.1 (RStudio, Boston, MA, USA) and SAS Enterprise Guide version 6.1 (SAS Institute Inc., Cary, NC, USA) were used for the statistical analyses.

## Results

**Figure 1:**
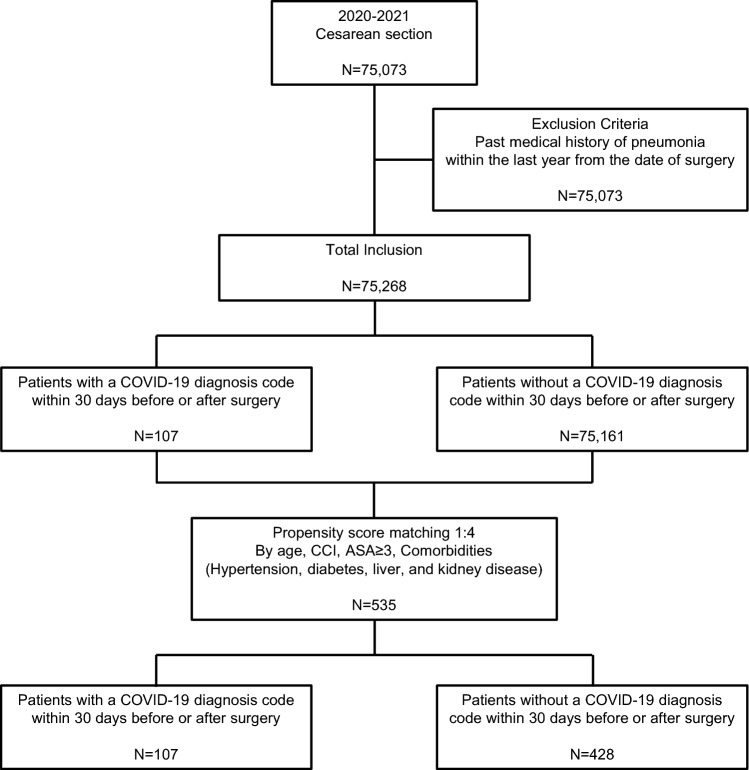
Flow diagram of study population.

We identified 75,703 patients who underwent cesarean section and were admitted to the hospital during 2020–2021. Among the total patients, 435 were excluded because a pneumonia diagnosis code was confirmed within 1 year prior to the surgery date. Finally, 75,268 patients were included in our study. Among them, 107 patients had a COVID-19 diagnosis code (U071) within 30 days before or after the surgery, while 75,161 patients did not have a COVID-19 diagnosis code (U071) within the same time frame (Fig. 1).

Table 1 depicts the baseline characteristics before and after PSM. Prior to PSM, baseline characteristics, including comorbid conditions, differed between the COVID (+) and COVID (−) groups. Following 1:4 PSM, 535 patients were included in each group, with no significant differences observed in covariates, including CCI, ASA physical status classification greater than 3, and comorbidities such as hypertension, diabetes, liver, and kidney disease.

**Table 1:** Baseline characteristics before and after propensity score matching.

Variable	Prematching					Postmatching				
	Total	COVID (+)	COVID (−)	p-value	SMD	Total	COVID (+)	COVID (−)	p-value	SMD
	(N = 75,268)	(N = 107)	(N = 75,161)			(N = 535)	(N = 107)	(N = 428)		
Year										
2020	17,805 (23.655)	53 (49.553)	17,752 (23.619)	< 0.0001		250 (46.729)	53 (49.553)	197 (46.028)	0.5158	
2021	57,463 (76.345)	54 (50.467)	57,409 (76.381)			285 (53.271)	54 (50.467)	231 (53.972)		
Age										
Under 18	24 (0.032)	0 (0)	24 (0.032)	0.9964	0.23855	0 (0)	0 (0)	0 (0)		0.00908*
18 and older	75,244 (99.968)	107 (100)	75,137 (99.968)			535 (100)	107 (100)	428 (100)		
Hospital type										
Tertiary Hospital	14,776 (19.631)	46 (42.991)	14,730 (19.598)	< 0.0001		145 (27.103)	46 (42.991)	99 (23.131)	< 0.0001	
General Hospital	19,234 (25.554)	47 (43.925)	19,187 (25.528)			169 (31.589)	47 (43.925)	122 (28.505)		
Clinic	41,258 (54.815)	14 (13.084)	41,244 (54.874)			221 (41.308)	14 (13.084)	207 (48.364)		
Hospitalization pathway										
Emergency	7961 (10.577)	27 (25.234)	7934 (10.556)	< 0.0001		80 (14.953)	27 (25.234)	53 (12.383)	0.0006	
Outpatient	67,160 (89.228)	80 (74.766)	67,080 (89.248)			454 (84.860)	80 (74.766)	374 (87.383)		
Others	147 (0.195)	0 (0)	147 (0.196)			1 (0.187)	0 (0)	1 (0.234)		
ASA 3 or higher	653 (0.868)	10 (9.346)	643 (0.855)	< 0.0001	0.39336	53 (9.907)	10 (9.346)	43 (10.047)	0.8282	0.02369*
Emergency surgery	773 (1.027)	1 (0.935)	772 (1.027)	0.3681		11 (2.056)	1 (0.935)	10 (2.336)	0.2356	
Night surgery	6424 (8.535)	18 (16.822)	6406 (8.523)	0.0021		72 (13.458)	18 (16.822)	54 (12.617)	0.2542	
Comorbidities										
History of Hypertension	1661 (2.207)	2 (1.869)	1659 (2.207)	0.2653	0.024	10 (1.869)	2 (1.869)	8 (1.869)	0.3048	0*
History of Diabetes	1605 (2.132)	5 (4.673)	1600 (2.129)	0.052	0.14065	27 (5.047)	5 (4.673)	22 (5.140)	0.1951	0.02163*
History of Liver disease	3626 (4.817)	18 (16.822)	3608 (4.800)	< 0.0001	0.39452	87 (16.262)	18 (16.822)	69 (16.121)	0.853	0.0189*
History of Kidney disease	479 (0.636)	2 (1.869)	477 (0.635)	0.1176	0.11117	10 (1.869)	2 (1.869)	8 (1.869)	0.9813	0*
History of CVA	4 (0.005)	0 (0)	4 (0.005)	0.9943		0 (0)	0 (0)	-	-	
Charson Comorbidity Index										
Continuous	0.1968	0.6262	0.1962	< 0.0001	0.48476	1	0.6262	1 (0.234)	0.8605	0.02096*
0	65,815 (87.441)	59 (55.140)	65,755 (87.486)	< 0.0001		299 (55.888)	59 (55.140)	299 (69.860)	0.3048	
1	6290 (8.357)	38 (35.514)	6252 (8.318)			182 (34.019)	38 (35.514)	182 (42.523)		
2	1933 (2.568)	7 (6.542)	1926 (2.562)			38 (7.103)	7 (6.542)	38 (8.879)		
3+	1230 (1.634)	3 (2.804)	1227 (1.632)			16 (2.991)	3 (2.804)	16 (3.738)		
Elixhauser's comorbidities										
Weight (continuous)	0.4166	1.1495	0.4155	< 0.0001		0.9944	1.1495	0.9556	0.1631	

Values are expressed as absolute numbers (percentages) or absolute numbers.

*SMD* standardized mean difference, *ASA* American Society of Anesthesiologists, *CVA* cerebrovascular accidents.

*SMD < 0.1 indicates effective matching between groups.

Table 2 presents the primary and secondary outcomes before and after the implementation of PSM. The primary outcome, which includes 30-day mortality, exhibited no significant differences between the two groups, both before and after matching. In the postoperative complication category, the COVID (+) group exhibited significantly higher rates of pneumonia, ARDS, PTE, and surgical site infection both before and after PSM. Hospital length of stay and admission costs were also significantly longer and higher, respectively, in the COVID (+) group before and after matching.

**Table 2:** Primary and secondary outcomes before and after propensity score matching.

Variable	Prematching				Postmatching			
	Total (n = 75,268)	COVID (+) (n = 107)	COVID (−) (n = 75,161)	p-value	Total (n = 535)	COVID (+) (n = 107)	COVID (−) (n = 428)	p-value
30-day mortality	19 (0.025)	0 (0)	19 (0.025)	0.9733	1 (0.187)	0 (0)	1 (0.234)	0.6667
Postoperative complication								
Pneumonia	330 (0.438)	11 (10.280)	319 (0.424)	< 0.0001*	13 (2.430)	11 (10.280)	2 (2.430)	< 0.0001*
ARDS	7 (0.009)	5 (4.673)	2 (0.003)	< 0.0001*	5(0.935)	5 (4.673)	0 (0)	0.0003*
PTE	89 (0.118)	5 (4.673)	84 (0.112)	< 0.0001*	7 (1.308)	5 (4.673)	2 (0.467)	0.0041*
Thromboembolic event	26 (0.035)	0 (0)	26 (0.035)	0.9637	1 (0.187)	0 (0)	1 (0.234)	0.8
Mechanical ventilation	1 (0.001)	0 (0)	1 (0.001)	0.9986	0 (0)	0 (0)	0 (0)	–
Cardiac arrest	16 (0.021)	0 (0)	16 (0.021)	0.9775	0 (0)	0 (0)	0 (0)	–
MI	22 (0.029)	1 (0.935)	21 (0.028)	0.0304	1 (0.187)	1 (0.935)	0 (0)	0.2
Surgical site infection	568 (0.755)	6 (5.607)	562 (0.748)	0.0002*	12 (2.243)	6 (5.607)	6 (1.402)	0.0147*
Sepsis	49 (0.065)	1 (0.935)	48 (0.064)	0.0651	1 (0.187)	1(0.935)	0 (0)	0.2
ARF	49 (0.065)	0 (0)	49 (0.065)	0.9326	1 (0.187)	0 (0)	1 (0.234)	0.8
Hepatic failure	15 (0.020)	0 (0)	15 (0.020)	0.9789	0 (0)	0 (0)	0 (0)	–
Hospitalization								
Hospitalization	75,239 (99.961)	99 (92.523)	75,140 (99.972)		527 (98.505)	99 (92.523)	428 (100.000)	
Hospital length of stay	6.3	8.9	6.3	< 0.0001*	7.14	8.98	6.715	0.001*
Hospitalization costs	2,908,489	5,556,379	2,905,000	0.001*	3,915,680	5,556,378	3,536,173	0.013*
ICU Admission								
ICU admission	41 (0.054)	2 (0.187)	39 (0.516)		3 (0.560)	2 (1.869)	1 (0.234)	
ICU length of stay	12.195	27	11.436	0.26	19.33	27	4	0.468
ICU costs	19,202,118	22,418,755,	19,037,162	0.867	21,879,060	22,418,755	20,799,670	0.954
ECMO implementation	10 (0.013)	0 (0)	10 (0.132)	0.986	0 (0)	0 (0)	0 (0)	

Values are expressed as absolute numbers (percentages) or absolute numbers.

*ARDS* acute respiratory distress syndrome, *PTE* pulmonary thromboembolism, *MI* myocardial infarction, *ARF* acute renal failure, *ICU* intensive care unit, *ECMO* Extracorporeal Membrane Oxygenation.

*p < 0.05 indicates statistically significant differences between groups.

Table 3 displays the results of logistic regression analysis examining overall pulmonary complications in the COVID (+) group compared to the matched COVID (−) group. In the COVID (+) group, the adjusted odds ratio (OR) for pulmonary complications was 79.772 with a 95% confidence interval (CI) of 11.059 to 575.44, showing a statistically significant increase (p < 0.001). Additionally, among patients with ASA physical status classification 3 or higher, the adjusted OR for pulmonary complications was 23.453 (95% CI 4.232–129.987, p = 0.0003), demonstrating statistical significance.

**Table 3:** Logistic regression analysis for overall pulmonary complications in the COVID (+) group compared with the matched COVID (−) group.

	Univariable		Multivariable	
	Crude OR (95% CI)	p-value	Adjusted OR (95% CI)	p-value
COVID group				
COVID (−)	1		1	
COVID (+)	34.728 (7.807–154.479)	< 0.0001	79.772 (11.059–575.44)	< 0.0001*
Year				
2020	1		1	
2021	2.14 (0.482–9.494)	0.3169	0.344 (0.049–2.434)	0.2849
Anesthesia				
General	1		1	
Regional	0.604 (0.227–1.612)	0.3144	2.049 (0.486–8.633)	0.3284
Hospital type				
Tertiary care hospital	1		1	
General hospital	0.98 (0.346–2.77)	0.1234	1.344 (0.388–4.656)	0.94
Clinic	0.18 (0.037–0.879)	0.0246	1.642 (0.261–10.337)	0.6906
ASA 3 or higher	7.183 (2.61–19.764)	0.0001	23.453 (4.232–129.987)	0.0003*
Comorbidities				
History of diabetes	1.183 (0.151–9.265)	0.873	0.893 (0.061–13.034)	0.934
History of liver disease	1.613 (0.513–5.067)	0.4134	1.644 (0.273–9.906)	0.5876
Charson Comorbidity Index				
0	1		1	
1	1.455 (0.519–4.082)	0.8922	1.066 (0.276–4.119)	0.6246
2	0.983 (0.12–8.083)	0.6867	0.465 (0.026–8.409)	0.5972
3+	2.426 (0.285–20.667)	0.4858	0.768 (0.025–23.263)	0.9847
Elixhauser's comorbidities				
Weight (continuous)	1.175 (0.849–1.625)	0.331		

Values are presented as odds ratio with corresponding 95% confidence intervals.

*OR* odds ratio, *CI* confidence interval, *ASA* American Society of Anesthesiologists.

In a subgroup analysis of anesthesia methods, there were no differences in 30-day mortality and postoperative complications after matching (Table 4). While pre-matching revealed certain disparities in complications and outcomes, post-matching analysis demonstrates a reduction in these differences, emphasizing the importance of accounting for potential confounding factors when evaluating the impact of anesthesia types on surgical outcomes among COVID (+) and COVID (−) patients.

**Table 4:** Subgroup analysis for mortality and complications according to anesthetic methods.

Variable	Prematching					p-value	Postmatching					p-value
	Total n = 75,268	General		Regional			Total n = 535	General		Regional		
		COVID + n = 53	COVID– n = 17,752	COVID + n = 54	COVID– n = 57,409			COVID + n = 53	COVID– n = 197	COVID + n = 54	COVID– n = 231	
30-day mortality	19	0	15	0	4	-	1	0	1	0	0	-
Postoperative complication												
Pneumonia	330	6	199	5	120	0.2099	13	6	1	5	1	0.5385
ARDS	7	4	2	1	0	0.7143	5	4	0	1	0	–
PTE	89	2	69	3	15	0.0489	7	2	2	3	0	0.2857
Thromboembolic event	26	0	18	0	8	–	1	0	1	0	0	–
Mechanical ventilation	1	0	1	0	0	–	0	0	0	0	0	–
Cardiac arrest	16	0	13	0	3	–	0	0	0	0	0	–
MI	22	1	14	0	7	0.6818	1	1	0	0	0	–
Surgical site infection	568	1	343	5	219	0.034	12	1	3	5	3	0.2424
Sepsis	49	1	34	0	14	0.7143	1	1	0	0	0	–
ARF	49	0	37	0	12	–	1	0	1	0	0	–
Hepatic failure	15	0	7	0	8	–	0	0	0	0	0	–

Values are expressed as absolute numbers.

*ARDS* acute respiratory distress syndrome, *PTE* pulmonary thromboembolism, *MI* myocardial infarction, *ARF* acute renal failure.

## Discussion

In this study, we investigated the impact of perioperative COVID-19 infection on obstetric anesthesia in patients undergoing cesarean section procedures. Utilizing a comprehensive dataset from the Korean NHIS, our findings shed light on crucial aspects of maternal care during the COVID-19 pandemic. The analysis, including propensity score matching, revealed significant differences in postoperative complications, hospital length of stay, and admission costs between the COVID (+) and COVID (−) groups. Notably, despite matching for various demographic and comorbidity factors, the COVID (+) group exhibited higher rates of pulmonary complications, emphasizing the need for targeted interventions and heightened vigilance during obstetric anesthesia in the context of COVID-19.

For our analysis, we defined the COVID (+) group as patients with a COVID-19 diagnosis code (U071) within 30 days before or after surgery. This definition was informed by CDC research indicating that most patients recover from acute COVID-19 illness within four weeks, allowing us to capture the immediate and short-term effects of the infection on surgical and anesthesia outcomes^15^. Additionally, prior research have shown that patients undergoing surgery within four weeks of a COVID-19 diagnosis have a significantly higher risk of pulmonary complications and increased mortality rates^16–18^. Given these considerations and the practical challenges of subdividing our dataset into pre- and post-surgery infections, we adopted a 30-day window to comprehensively evaluate the impact of COVID-19 during this critical period.

According to the pre-matching baseline characteristics in our study, the rate of admissions through the emergency department was 25.234% in the COVID (+) group, compared to 10.556% in the COVID (−) group, indicating more than a two-fold increase. Furthermore, the rates of emergency surgeries and nighttime surgeries were approximately twice as high in the COVID (+) group. Supporting these findings, a retrospective cohort study conducted in Australia reported a 2.3% increase in the emergency cesarean section rate during the COVID-19 pandemic compared to the pre-pandemic period^19^. The CCI in our pre-matching baseline data was significantly higher in the COVID (+) group, indicating a greater burden of comorbidities. Although the prevalence of diabetes was higher in the COVID (+) group at 4.673% compared to 2.129% in the COVID (−) group, this difference did not reach statistical significance. Similarly, Zanardo et al. reported a significantly higher prevalence of gestational diabetes mellitus during the 2020 COVID-19 pandemic compared to the pre-pandemic period^20^.

Recent multicenter propensity score-matched studies, revealing that COVID-19 in pregnancy is associated with an increased risk of disease severity, including hospitalization, ICU admission, the use of vasoactive agents, oxygen supplementation, invasive mechanical ventilation, and death^2,21^. As COVID-19 pneumonia rapidly progresses from focal to diffuse consolidation of lung parenchyma, the limited total lung capacity at term, due to diaphragmatic pressure from the growing uterus, might render pregnant women more susceptible to hypoxemic respiratory failure, leading to increased morbidity^6^. Our study aligns with previous research, indicating that pregnant individuals with COVID-19 infection face an increased risk of complications including pneumonia, ARDS, PTE, surgical site infections, and hospitalization. This observation may also be associated with physiological changes during pregnancy, such as elevated heart rate and oxygen consumption, a deviation from cell-mediated immunity, and an elevated susceptibility to thromboembolic disease^5,7^. These findings emphasize the necessity of continued vigilance and tailored medical care for pregnant patients during the pandemic. Healthcare providers should maintain a high index of suspicion for COVID-19 in this population, closely monitor for potential complications, and consider strategies to mitigate risks. In a nationwide prospective cohort propensity score-matched analysis conducted in Mexico, the study reported that pregnancy is identified as a risk factor for death, pneumonia, and ICU admission in SARS-CoV-2-infected women of reproductive age^22^. The World Association of Perinatal Medicine Working Group reported a maternal mortality risk of 0.8% in pregnancies complicated by SARS-CoV-2 infection^23^. Consistent with this finding, our cohort observed an overall maternal mortality 0.935% in the COVID (+) group. Additionally, a Korean nationwide population-based cohort study analyzing cause of maternal mortality from 2003 to 2018 reported a six-week maternal mortality rate of 0.019% associated with cesarean sections^24^. In contrast, our analysis during the COVID-19 pandemic showed a higher mortality rate of 0.025%. Although an increase in cesarean section-associated maternal mortality was observed during the COVID-19 pandemic, our study did not find a significant difference in mortality between the COVID (+) group and the COVID (−) group. This is likely due to the very low number of mortality cases observed^25^. Including data from the entire pandemic period, up to 2023, might have provided more cases, enhancing the statistical significance.

Prior international studies have reported high cesarean delivery rates among women with SARS-CoV-2 infection. Notably, a nationwide cross-sectional study from Korea^26^ found a rate of 78.1%, a case series from China^27^ reported 76.9%, and cohort studies from the United Kingdom^28^ and Spain^29^ observed rates of 59% and 47%, respectively. This observation suggests a significant association between COVID-19 infection during pregnancy and an increased preference for cesarean delivery. In the early stages of the global COVID-19 pandemic (SARS-CoV-2), guidelines endorsed prioritizing regional anesthesia over general anesthesia for cesarean sections in medical practice^30,31^. To minimize the aerosolization of viral particles during endotracheal intubation/extubation and airway manipulation, neuraxial anesthesia is the preferred choice^8^. The COVID-19 pandemic has led to a notable decline in the use of general anesthesia for cesarean sections. In the UK, the rates of general anesthesia for these procedures have decreased significantly^32^. Prior to the pandemic, the rate was 7.5%, which dropped to 3.3% in 2020, representing a significant reduction of 4.2% (95% CI 1.7–6.6; p = 0.0016) (p = 0.0042). Similarly, the group in Israel has reported an increase in the use of neuraxial anesthesia for planned cesarean sections, from 44.8% to 79.3% (p < 0.0001)^33^. A cross-sectional study in the Northwest of England, involving more than 17,000 births during the COVID-19 pandemic, found higher rates of neuraxial anesthesia and a significant reduction in conversions to general anesthesia. General anesthesia rates dropped from 7.7 to 3.7%^34^. Contrastingly, Katz et al. reported that symptomatic SARS-CoV-2-infected patients are more prone to receiving general anesthesia for cesarean delivery (adjusted OR 3.69; 95% CI 1.40–9.74) as a result of maternal respiratory failure^9^. Concerns about performing neuraxial anesthesia in COVID-19-positive parturient may arise due to the potential risk of systemic infection, even though infectious complications are relatively uncommon. According to a study involving pregnant women with SARS-CoV-2 undergoing cesarean section and cerebrospinal fluid (CSF) analysis during spinal anesthesia, the genomes of SARS-CoV-2 and other neurotropic viruses were not detected in any samples^35^. Therefore, based on these findings, spinal anesthesia was deemed safe for SARS-CoV-2-positive pregnant women with mild disease. In addition, According to a prospective observational study conducted by Ababneh et al., based on a cohort of 62 subjects, spinal anesthesia was reported to be safe for COVID-19 positive parturient in terms of hemodynamic stability and perioperative complications^36^. Until now, there has been no meaningful study comparing outcomes based on anesthesia methods (general anesthesia vs. neuraxial anesthesia) in cesarean deliveries for mothers infected with COVID-19. In our study, we conducted a subgroup analysis, adjusting for comorbidities, including the CCI, ASA physical status classification greater than 3, and conditions such as hypertension, diabetes, liver, and kidney disease. The results did not reveal significant differences in outcomes based on the chosen anesthesia method. Various confounding factors, such as fetal distress, deterioration of the mother’s health, catheter failure, and inappropriate timing of LMWH for regional anesthesia, contribute to the preference for general anesthesia over neuraxial anesthesia, making outcome comparisons challenging^37^.

A hospital-based prospective study conducted at 12 centers across 9 countries reported that emergency caesarean delivery increases the risk of maternal deaths compared to elective cesarean delivery^38^. There is no available data on the mortality comparison between elective and emergency cesarean delivery in COVID-19-infected mothers. In our study, regression analysis revealed an adjusted OR of 22.281 for cases where emergency surgery was performed, with a 95% CI ranging from 1.363 to 364.113. However, the considerable length of the confidence interval suggests a somewhat unstable outcome. This is attributed to the low absolute number of actual mortality cases.

Ensuring the safety of both mother and fetus is paramount in obstetric anesthesia during cesarean section delivery. The challenges posed by COVID-19 in surgical and anesthesia scenarios are concerning, potentially impacting the overall health of both mother and fetus. This study reveals no significant difference in 30-day mortality between COVID-positive and COVID-negative patients, irrespective of the anesthesia type. It suggests that while COVID-19 infection increases the risk of specific complications such as pneumonia, ARDS, PTE, and surgical site infections, it does not directly influence short-term mortality in this context.

These findings offer reassurance to clinicians and pregnant patients facing challenging decisions regarding cesarean delivery amid the pandemic. However, it's crucial to acknowledge that the absence of a difference in short-term mortality does not diminish the increased risk of complications associated with COVID-19.

Given the evolving nature of the COVID-19 pandemic and the emergence of new variants, ongoing research is crucial. Future studies should focus on long-term maternal and fetal outcomes, the effectiveness of different anesthesia techniques in the COVID-19 context, and the impact of vaccination status on surgical and obstetric outcomes. Moreover, research exploring the psychosocial impact of the pandemic on pregnant individuals undergoing cesarean sections would be valuable.

Despite the valuable insights offered by this study, it is crucial to consider several limitations. Firstly, the inherent nature of retrospective claim data, primarily crafted for reimbursement purposes rather than clinical research, needs acknowledgment^39^. Consequently, critical clinical information, such as patients' clinical data or disease severity, is not included in the database. Secondly, the study's findings are based on a specific timeframe (2020–2021), and the dynamics of the pandemic may have evolved since then. Additionally, long-term outcomes beyond 30 days were not assessed, and further research is warranted to explore the potential impact of COVID-19 on maternal and fetal health in the extended postoperative period. Thirdly, due to the limitations of our dataset, we could not distinguish between COVID-19 infections occurring before and after cesarean section, which could have provided more precise insights into the distinct impacts during the acute and recovery phases of the infection. Lastly, the PSM process significantly reduced the sample size, leading to increased variability and wider CIs in the multivariable analysis. In addition, the inability to assess colinearity among the variables may further contribute to these large CIs. Future studies with larger sample sizes and through check for colinearity may help to reduce the variance and provide more stable estimates.

## Conclusions

In summary, our study found that COVID-19 infection had little impact on 30-day mortality rates in patients undergoing cesarean section. However, patients with COVID-19 exhibited a significantly higher incidence of postoperative complications, including pneumonia, acute respiratory distress syndrome (ARDS), pulmonary thromboembolism (PTE), surgical site infections, and prolonged hospitalization. The review of outcomes during the COVID-19 pandemic period emphasizes the critical need for increased surveillance and management of pulmonary complications, particularly in patients with pre-existing comorbidities and an ASA score greater than 3.

### Supplementary Information

Supplementary Figure 1.

## Data Availability

The datasets used and analyzed during the current study are available from the corresponding author on reasonable request. Requests for access to the data should be directed to Si Ra Bang at sira1045@naver.com, who will facilitate the process in compliance with the necessary permissions from the National Health Insurance System. It is important to note that the data were used under a specific license for this study and are subject to restrictions related to their accessibility. Further information on how to request these data can be found at the National Health Insurance Sharing Service website (https://nhiss.nhis.or.kr/bd/ab/bdaba000eng.do). The provision of data will depend on the approval of the National Health Insurance System and adherence to the terms of the license under which the data were used.
