# Supplementary material for: Impact of perioperative COVID-19 infection on postoperative complication in cesarean section using Korean National Health insurance data
Source: Sci Rep. 2024 Jul 11;14:16001. doi: 10.1038/s41598-024-66901-5 (PMC11237102; doi:10.1038/s41598-024-66901-5)

**Supplementary Figure 1. Standardized Mean Differences (SMD) of Baseline Characteristics Before and After Propensity Score Matching (PSM).** The plot displays the standardized mean differences (SMD) of baseline characteristics before and after propensity score matching (PSM). The x-axis represents the absolute standardized difference (ASD) values, and the y-axis lists the baseline characteristics, including age, Charlson comorbidity index (CCI), American Society of Anesthesiologists (ASA) classification, hypertension, diabetes, liver diseases, and kidney diseases. The blue line with dots indicates the SMD before PSM, showing significant imbalances in covariates with ASD values up to 0.5. The red line with dots represents the SMD after PSM, illustrating improved balance across covariates with ASD values below the threshold of 0.1, indicating successful matching. The dashed vertical line at 0.1 represents the threshold below which covariates are considered well-balanced.


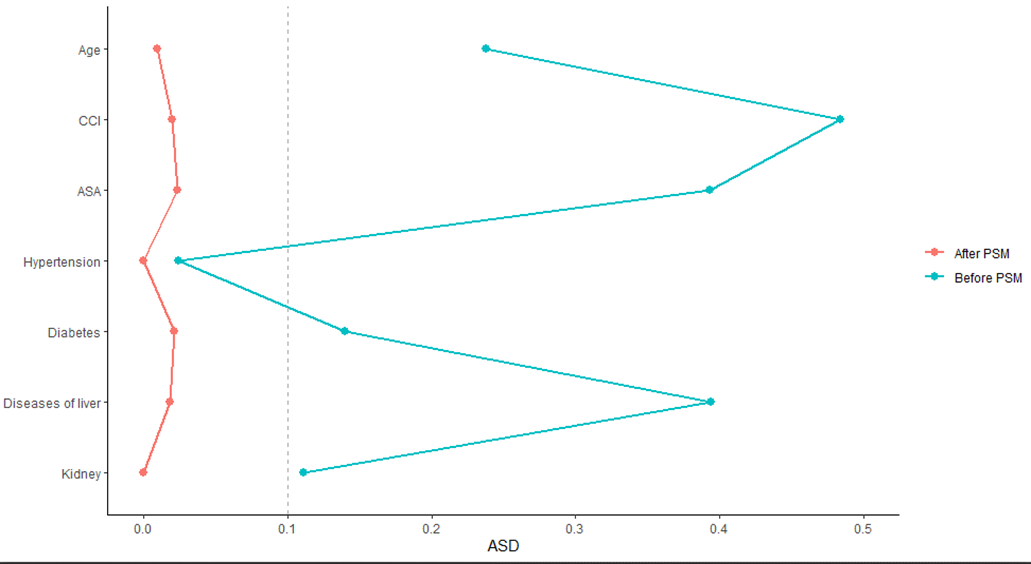

Supplement: Supplementary file 1 — Supplementary Figure 1. [file 41598_2024_66901_MOESM1_ESM.docx]
